# Halotolerant microbial consortia able to degrade highly recalcitrant plant biomass substrate

**DOI:** 10.1007/s00253-017-8714-6

**Published:** 2018-02-03

**Authors:** Larisa Cortes-Tolalpa, Justin Norder, Jan Dirk van Elsas, Joana Falcao Salles

**Affiliations:** Department of Microbial Ecology, Groningen Institute for Evolutionary Life Sciences, University of Groningen, Nijenborgh 7, 9747 AG Groningen, The Netherlands

**Keywords:** Biomass degradation, Halotolerant degrader consortia, Bacterial-fungal consortia

## Abstract

The microbial degradation of plant-derived compounds under salinity stress remains largely underexplored. The pretreatment of lignocellulose material, which is often needed to improve the production of lignocellulose monomers, leads to high salt levels, generating a saline environment that raises technical considerations that influence subsequent downstream processes. Here, we constructed halotolerant lignocellulose degrading microbial consortia by enriching a salt marsh soil microbiome on a recalcitrant carbon and energy source, i.e., wheat straw. The consortia were obtained after six cycles of growth on fresh substrate (adaptation phase), which was followed by four cycles on pre-digested (highly-recalcitrant) substrate (stabilization phase). The data indicated that typical salt-tolerant bacteria made up a large part of the selected consortia. These were “trained” to progressively perform better on fresh substrate, but a shift was observed when highly recalcitrant substrate was used. The most dominant bacteria in the consortia were *Joostella marina*, *Flavobacterium beibuense*, *Algoriphagus ratkowskyi*, *Pseudomonas putida*, and *Halomonas meridiana.* Interestingly, fungi were sparsely present and negatively affected by the change in the substrate composition. *Sarocladium strictum* was the single fungal strain recovered at the end of the adaptation phase, whereas it was deselected by the presence of recalcitrant substrate. Consortia selected in the latter substrate presented higher cellulose and lignin degradation than consortia selected on fresh substrate, indicating a specialization in transforming the recalcitrant regions of the substrate. Moreover, our results indicate that bacteria have a prime role in the degradation of recalcitrant lignocellulose under saline conditions, as compared to fungi. The final consortia constitute an interesting source of lignocellulolytic haloenzymes that can be used to increase the efficiency of the degradation process, while decreasing the associated costs.

**Electronic supplementary material:**

The online version of this article (10.1007/s00253-017-8714-6) contains supplementary material, which is available to authorized users.

## Introduction

Lignocellulosic plant biomass is the most abundant global carbon source. Aside its availability and low cost, its utilization can attenuate the conflict between food and energy crops (Kinet et al. 2015). However, the main obstacle in its widespread application is the high cost of the pretreatments, which are necessary to open the intricate polysaccharide structure. Such pretreatments enhance the accessibility of enzymatic attack (Talebnia et al. 2010) and decrease the proportion of crystalline cellulose and lignin content, the two main causes of the recalcitrance of lignocellulose. Overcoming this recalcitrance is fundamental for getting access to the polymers that yield sugar monomers, which can be transformed in valuable compounds such as sustainable biomaterials, biofuel, and biochemicals (Khoo et al. 2016).

In the past years, three different pretreatment processes have been proposed to improve the digestibility of lignocellulose materials. These aimed to foster (1) the degradation of hemicellulose, by acid or hot water treatment, (2) that of lignin, by alkaline pretreatment to break the lignin-carbohydrate linkage bond, and (3) the generic disruption of the matrix by thermal treatment (Brethauer and Studer 2015). Such pretreatments not only increase the global cost of the bioprocess but also generate diverse compounds that interfere with downstream processes (Jönsson and Martin 2016; Rabemanolontsoa and Saka 2016).

A promising new pretreatment method is based on the application of ionic liquids (ILs), organic salts (“green solvents”) (Sun et al. 2016) that are liquid at room temperature. Using ILs, lignocellulose biomass is exposed to highly saline conditions that disrupt the rigid lignocellulose structure, leading to a considerable reduction in cristallinity and increased accessibility to enzymatic attack. However, when using acid/base treatment or ILs, subsequent enzymatic hydrolysis of the substrate can only be performed after several washing steps aiming at salt removal, as salt often inhibits enzymatic activity. The use of haloenzymes (or enzymes tolerant to high salinity) (Gunny et al. 2014) could represent a sound alternative strategy to increase the efficiency and reduce the cost of the bioprocess.

Dilution-to-stimulation has been used as a successful method to enrich microbial consortia capable of degrading plant biomass and their respective enzymes (Brossi et al. 2015; Maruthamuthu et al. 2016). These consortia have been obtained from a variety of sources (Cortes-Tolalpa et al. 2016) and are often capable of degrading a range of lignocellulose materials (Okeke and Lu 2011; Brossi et al. 2015). For instance, we have shown that consortia obtained from different microbial sources naturally enriched in lignocellulose material quickly reach a stabilization phase (phase of relative stability of the consortium in terms of composition and activity) during the enrichment process (Cortes-Tolalpa et al. 2016). Although the various consortia did not differ in their final degradation potential, they reached this through different activities, as they differed in their enzymatic pools. Thus, the source of the inoculum used for the enrichment clearly influenced the final outcome and type of process. Despite the success of this approach, which leads to consortia capable of “attacking” or consuming the most labile part of the substrate, these consortia have been obtained under “low” salt concentrations. Given the importance of the microbial source, the development of such consortia using halotolerant microbes could provide an interesting perspective.

The aim of this study was to examine whether it is possible to obtain a halotolerant microbial consortium capable of degrading lignocellulose biomass (raw wheat straw) at high rate under high-salt conditions. For that, we used as inoculum the microbial community obtained from salt marsh soil from a the island of Schiermonnikoog, the Netherlands. This was previously found to be adapted to high-salt concentrations and to harbor key genes involved in lignocellulose degradation (Dini-Andreote et al. 2014; Wang et al. 2016). In addition, to generate consortia with high degradation potential under high-salt conditions, selection on pre-digested recalcitrant substrate was applied.

## Methods

### Culture media and lignocellulose substrate

For the experiment, we used a the mineral medium solution MMS (7 g/L Na_2_HPO_4_·2H_2_O; 2 g/L K_2_HPO_4_; 1 g/L (NH_4_)_2_SO_4_; 0.1 g/L Ca (NO_3_)_2_·4H_2_O; 0.2 g/L MgCl_2_·6H_2_O g/L, pH 7.2) (Cortes-Tolalpa et al. 2016), supplemented with 25 g per liter of NaCl. The medium was further supplemented with vitamin solution (0.1 g Ca-pantothenate, 0.1 g cyanocobalamine, 0.1 g nicotinic acid, 0.1 g pyridoxal, 0.1 g riboflavin, 0.1 g thiamin, 0.01 g biotin, 0.1 g folic acid; H_2_O 1 L) and trace metal solution (2.5 g/L EDTA; 1.5 g/L FeSO_4_; 0.025 g/L CoCl_2_; 0.025 g/L ZnSO_4_; 0.015 g/L MnCl_2_; 0.015 g/L NaMoO_4_; 0.01 g/L NiCl_2_; 0.02 g/L H_3_BO_3_; 0.005 g/L CuCl_2_). “Raw wheat straw” used as lignocellulose source, was air-dried (50 °C) before cutting it into pieces of about 5 cm length and then the pieces were thoroughly ground, using a mill hammer, to pieces ≤ 1 mm. No pre-treatment was performed (untreated raw substrate). Sterility of the substrate was verified following plating on trypticase soy agar (TSA) plates. All chemicals and reagents used in this work were of analytic molecular biology grade (Sigma-Aldrich, Darmstadt, Germany).

### Sample collection

The source of the microbial community used in this experiment was soil from Schiermonnikoog island (53°29’ N 6°10′ E), 10-g of surface soil (0–10 cm) representative of the 105-year old plot located at the end of the natural primary succession observed in this island (Wang et al. 2016), the soil samples were thoroughly mixed. These soils are characterized by pH varying from 7.4–7.6 and sodium concentration from 3541 ± 170 to 5188 ± 624 mg dm^−3^, depending on the period of the year (Dini-Andreote et al. 2014). Cell suspension was prepared by adding 10 g of the soil to 250 mL flasks containing 10 g of sterile gravel in 90 mL of MMS. The suspension was shaken for 30 min at 200 rpm (room temperature).

### Enriched consortia

To start the enrichment, 250 μL of the suspension was added to each of triplicate 100-ml Erlenmeyer flasks containing 25 mL of MMS supplemented with 1% (*w*/*v*) sterilized wheat straw, 25 μL of vitamin and 25 μl of trace metal solution. Flasks were incubated at 28 °C, with shaking at 180 rpm. Cultures were monitored by counting cells in a Bürker-Türk chamber every day. Experiments started with around 5 log cells/mL. Once the systems had reached around 9 log cells/mL (and straw had visually been degraded), 25 μL of culture was transferred to 25 mL of fresh medium (dilution 10^−3^). During the first part of the enrichment, from transfer one to six—the adaptation phase—we used fresh wheat straw. In the second part of the experiment, from transfer seven to ten—the stabilization phase—we used recalcitrant wheat straw. This consisted of the sterilized substrate recovered at the end of the adaptation phase (transfers five and six), partially consumed by microbial consortia, and therefore encompassing only the most recalcitrant structure of the substrate (Supplemental Fig. S1). Following each transfer (T), part of the bred consortia was stored in 20% glycerol at − 80 °C. The consortia of the T1, T3, T6, T7, and T10 flasks were used for all subsequent analyses, as detailed below. As controls, we used microbial sources in MMS without substrate (CA 1, 2, 3) as well as MMS plus substrate without inoculum (CB 1, 2, 3). Before starting the enrichment Erlenmeyer flasks containing 25 mL lignocellulose, media were autoclaved at 121 °C for 27 min.

### DNA extraction

One mL of selected cultures was used for community DNA extraction using the “Power Soil” DNA extraction kit (inoculum source) (MoBio® Laboratories Inc., Carlsbad, USA) and the UltraClean DNA Isolation Kit (each enriched consortium and isolates). The instructions of the manufacturer were followed, except that the resuspension of the DNA from the inoculum sources was in 60 μL resuspension fluid.

### PCR followed by denaturing gradient gel electrophoresis (PCR-DGGE)

Total community DNA was used as the template for amplification of the partial 16S rRNA gene fragment by PCR with primers F968 with a GC clamp attached to the 5′-end and universal bacterial primer R1401.1b. For ITS1 amplification, primers EF4/ITS4 were used; this PCR was followed by a second amplification with primers ITS1f-GCITS2. Primer sequences, the reaction mixtures, and cycling conditions have been described (Brons and van Elsas 2008; Pereira e Silva et al. 2012). The DGGE was performed as reported by Cortes-Tolalpa et al. (2016). The DGGE patterns were then transformed to a band-matching table using GelCompar II software (Applied Maths, Sint Martens Latem, Belgium).

### Quantitative PCR (q-PCR)

The 16S rRNA gene region V5-V6 (bacteria), as well as the ITS1 region (fungi), were amplified using 1 ng of community DNA as the template and primers 16SFP/16SRP and 5.8S/ITS1 (Pereira e Silva et al. 2012), respectively. Standard curves were constructed using serial dilutions of cloned 16S rRNA gene and ITS1 fragments from *Serratia plymuthica* (KF495530) and *Coniochaeta ligniaria* (KF285995), respectively. The gene target quantification was performed, in triplicate, in an ABI Prism 7300 Cycler (Applied Biosystem, Lohne, Germany).

### Bacterial community sequencing and analyses

Amplicons of 250 bp were generated based on primers amplifying the V4-V5 of the 16S rRNA gene. PCR amplifications were conducted in triplicate reactions for each of the 18 samples with the 515F/806R primer set (Supplemental Table S1). PCR and sequencing were performed using a standard protocol (Caporaso et al. 2012). Illumina MiSeq sequencing was performed at GENEWIZ (South Plainfield, USA). We processed the raw data using the “quantitative insight into microbial ecology” (QIIME) software, version 1.91. The sequences were de-multiplexed and quality-filtered using split_libraries_fastq.py default parameters (Bokulich et al. 2013). The derived sequences were then clustered into operational taxonomic units (OTUs) using open-reference OTU picking against the Greengenes reference OTU data base with a 97% similarity threshold (Rideout et al. 2014). Then, we performed quality–filtering to discard OTUs present at very low abundance (< 0.005%) of the total number of sequences (Bokulich et al. 2013). An even sampling depth of 20,000 sequences per sample was used for assessing α- and β-diversity measures. Metrics for α-diversity were Chao1 index (estimated species richness) and Shannon index (quantitative measure of species). β-diversity analyses among the final consortia were performed using unweighted UniFrac distance matrix. Matrix similarity, PERMANOVA, and principal coordinate analyses (PCA), were performed by using phyloseq (McMurdie and Holmes 2013). Differential OTU abundance was calculated using DESeq2 with phyloseq (Supplemental Fig. S2) (Love et al. 2014; Mcmurdie et al. 2014). The comparison was made between sequential transfers (inocula-T1, T1-T3, T3-T6, T6-T7, T7-T10) and between the two main phases, adaption and stabilization phase, respectively.

### Isolation and identification of bacterial and fungi

From transfers 6 and 10, we isolated bacterial and fungal strains, using R2A (BD Difco®, Detroit, USA) and potato dextrose agar (PDA) (Duchefa Biochemie BV, Haarlem, The Netherlands), respectively. The isolation part can be found in Electronic supplemental material 1 (ESM 1). The primer pair U1406R and B8F was used for amplification of the 16S rRNA gene of bacterial strains, in the following PCR: initial denaturation at 95 °C for 5 min; 35 cycles of 95 °C for 1 min, 52 °C for 30 s, 72 °C for 2 min and final extension at 72 °C for 7 min. For identification of fungal strains the primers EF4 and ITS4 were used for amplification of the ITS1 region of the 18S rRNA gene, according to the following PCR : initial denaturation at 95 °C for 5 min; 34 cycles of 94 °C for 30 s, 55 °C for 30 s 72 °C for 1 min 30 s and final extension at  72 °C for 5 min. The amplicons were sequenced by Sanger technology (LGC Genomics, Lückenwalde, Germany) and the sequence of the PCR product was further used for bacterial and fungal identification. Taxonomic assignments of the sequences were done using BLAST-N (http://blast.stva.ncbi.nlm.nih.gov/Blast.cgi). We used the best BLAST hit affiliation for taxonomic assignment with a cutoff of 97 and 95% of identity of bacteria and fungi, respectively, and 95% of coverage. Sequences are publicly available in the GenBank database under accession numbers MF619963 to MF620009 (Tables 3 and 4). The recovered strains have been deposited in the German Collection of Microorganisms and Cell Cultures (DSMZ, Braunschweig, Germany).

### Matching bacterial strains with abundant OTUs

The recovered bacterial strains were linked to the OTUs based on sequence similarity. The almost-full-length 16S rRNA gene sequences from the strains were compared—in the specific V4-V5 region—to the sequences of the abundant OTUs using ClustalW. Phylogenetic analyses (*pairwise *distance) were conducted with MEGA v6 (Tamura et al. 2013) using Maximum Likelihood evolutionary distances that were computed using the Kimura-2 parameter method. The branch node strengths were tested with bootstrap analyses (1000 replications).

### Screening of lignocellulolytic enzyme production in recovered bacterial strains

Cellulases and hemicellulases in bacterial strains were detected by model substrate coupled to chromogenic compounds. The compounds 5-bromo-4-chloro-3-indolyl α-D-glucopyranoside (X-glu), 5-bromo-4-chloro-3-indolyl β-D-cellobioside (X-cell), 5-bromo-4-chloro-3-indolyl α-D-mannopyranoside (X-man), 5-bromo-4-chloro-3-indolyl β-D-galactopyranoside (X-gal), 5-bromo-4-chloro-3-indolyl β-D-xylopyranoside (X-xyl), and 5-bromo-4-chloro-3-indolyl β-fucopyranoside (X-fuc) (Sigma-Aldrich, Darmstadt, Germany) were used to detect the production and activity of α-glucosidase, cellobiohydrolases, α-mannosidase, β-galactosidase, β-xylosidase, and α-fucosidase enzymatic activity, respectively (Cortes-Tolalpa et al. 2016). The strains were spread in duplicate on R2A plates containing 1 M NaCl and each one of the chromogenic compounds listed above. The plates were incubated for 48 h at 28 °C. A positive enzymatic activity was observed as a blue colony growing on the plate.

### Lignocellulose degradation by selected halotolerant consortia

The final microbial consortia from transfers 1, 3, 6, 7, and 10 were incubated with 1% (*w*/*v*) mulched wheat straw under the culture condition that was previously described. After incubation, the final remaining particulate wheat straw was recovered from the microcosm flasks; the substrate was washed to remove microbial cells and sieved to obtain the degraded particles. The degradation rates of the components of the substrate, before and after incubation, were determined by Fourier-transformed infrared (FTIR) spectra (Adapa et al. 2011; Xu et al. 2013). All FTIR measurements were carried out on oven-dried material (50 °C, 24 h). Thirty-two scans were run per sample; all spectra between 800 and 1800 cm^1^ were used for the analyse (Krasznai et al. 2012). Each sample (calibration and consortium samples) was analyzed in triplicate. All spectra were subjected to baseline correction and then corrected for physical effects by second derivative Savitzky-Golay treatment (FitzPatrick et al. 2012). Correction and analysis using partial least squares (PLS) regression were conducted using Unscrambler X v.10 (CAMO, Woodbridge, USA). A mathematical model was created on the basis of a calibration with standard mixtures, consisting of hemicellulose (proxy beechwood xylan, ≥ 90%, Sigma-Aldrich, Steinheim, Germany), cellulose (powder, D-516, Macherey-Nagel, Düren, Germany) and lignin (alkaline, Sigma-Aldrich, Steinheim, Germany) in the proportion described in Supplemental Table S2 (Adapa et al. 2011). The model displayed *R*^2^ values of 0.9876, 0.9889, and 0.9763 and a slope of 0.9788, 1.000, and 0.9987 for hemicellulose, cellulose, and lignin, respectively. These models were then used to infer the proportion of each component in the samples (FitzPatrick et al. 2012; Krasznai et al. 2012). Finally, the degradation of hemicellulose, cellulose, and lignin was estimated by subtracting the percentage of the residual substrate from the total percentage of each hemicellulose component before degradation. Degradation rate was calculated using the following equation: $\frac{Ci- Cf}{Ci}x100$, where C_i_ is the total amount of compound before degradation and C_f_ is the residual component after degradation (Wang et al. 2011).

### Statistical analyses

One-way analysis of variance (ANOVA) followed by Tukey HSD pairwise group comparisons was performed in IBM SPSS Statistics version 24 (SPSS Inc., Chicago, USA).

## Results

### Halotolerant lignocellulolytic consortia are capable of degrading lignocellulose biomass under high-salt conditions

**Fig. 1:**
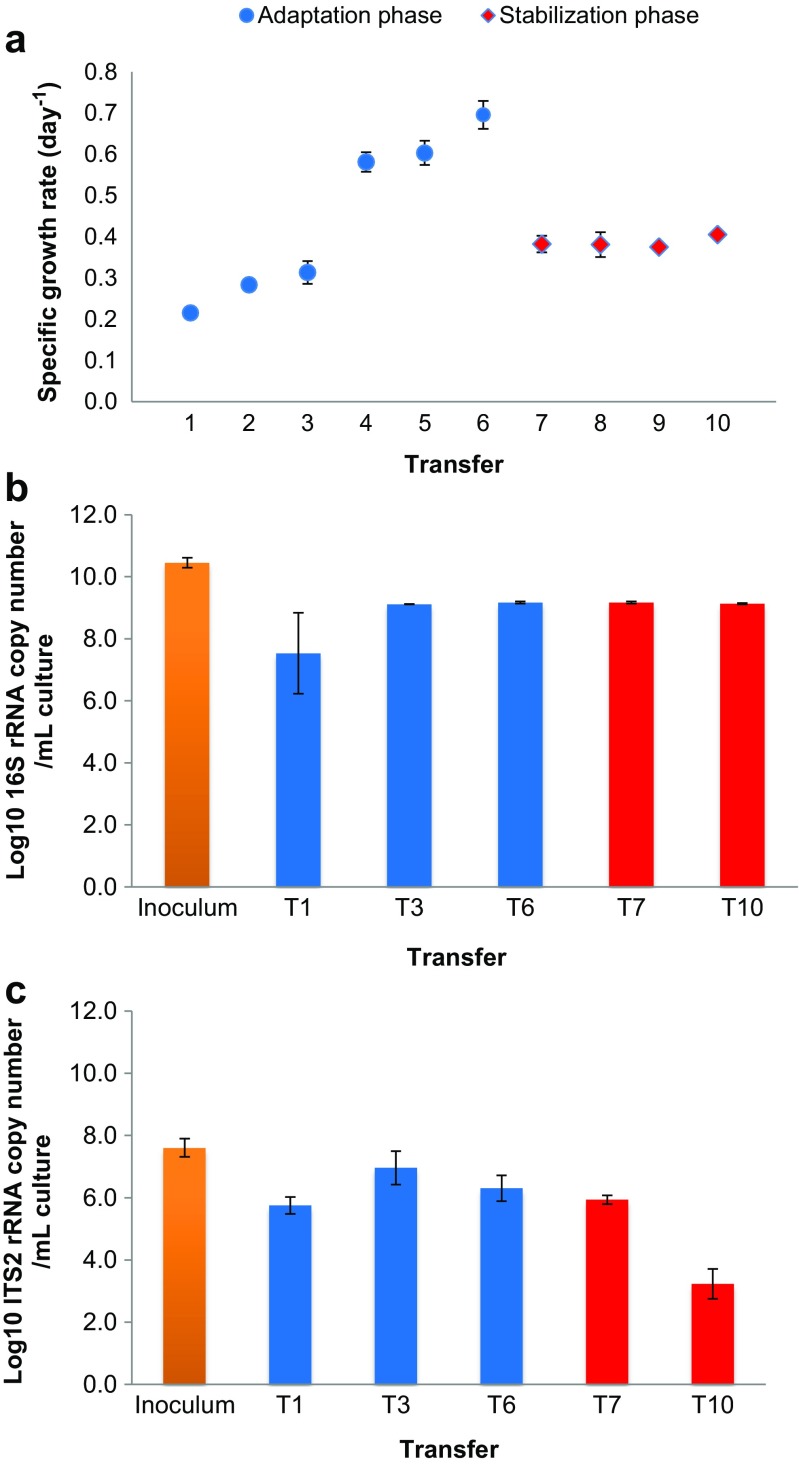
Microbial growth rates and abundances during the enrichment. **a** *Specific growth rate µ* (day^−1^) of microbial communities across the enrichment processes, as determined by microscopic cell counts. **b** *Bacterial abundances* during the enrichment (log copies per mL), as determined by qPCR targeting the 16S rRNA gene. **c** *Fungal abundances* during the enrichment (log copies per mL), as determined by qPCR targeting the ITS1 region. Yellow bars—original soil inoculum; blue circles and bars—adaption phase using fresh lignocellulose substrate (transfer 1 to 6); red diamonds and bars—stabilization phase using pre-digested substrate (transfer 7 to 10). Bars refer to standard errors of the mean (*n* = 3)

The microbial community from the salt marsh soil, used as the inoculum, was able to adapt to, and grow on, wheat straw as the single carbon and energy source and under saline conditions. Using microscopic counts, we found that, during the adaptation phase, from transfer one to six, the cultures exhibited a progressively increasing fitness, as indicated by an increasing specific growth rate over time. The average specific growth rate µ (h^−^^1^; ± standard deviation; see Fig. 1a) increased from 0.22 h^−1^ (± 0.01) to 0.70 h^−1^ (± 0.03), from T1 to T6. In the stabilization phase, we observed an almost twofold reduction in the growth rate immediately after substrate change, which dropped from 0.70 h^−1^ (±0.03) to 0.38 h^−1^ (±0.02) (Fig. 1a, see T6 and T7), after which it remained constant until the end of the experiment (T10). The reduced apparent fitness of the consortia was thus related to the increased recalcitrance of the substrate.

The microscopic cell counts were corroborated by the 16S rRNA gene and ITS1 copy numbers determined by qPCR, which were used as proxies for bacterial (Fig. 1b) and fungal community density (Fig. 1c), respectively. At the end of each transfer in the adaptation phase, the consortia reached maximal bacterial levels of (log scale): 7.5 ± 1.3 (T1), 9.1 ± 0.002 (T3), and 9.2 ± 0.034 (T6) (average log 16S rRNA gene copies per mL ± standard deviation). In the stabilization phase, these values were similar: 9.2 ± 0.034 (T7) and 9.1± 0.02 (T10). The fungal abundances (measured by numbers of ITS1 gene copies) at the end of transfers 1, 3, 6, and 7 reached around (log scale) 6 per mL. However, we observed a significant reduction of ITS1 copies in the stabilization phase, from T7 to T10 (*T* test, *P* < 0.05), indicating that under saline conditions, fungi were strongly deselected by the increase of substrate recalcitrance.

### Shifts in bacterial and fungal community composition

The microbial consortia were first analyzed by bacterial- as well as fungal-specific PCR-DGGE to examine the overall changes in community composition in selected transfers. Multidimensional scaling (MDS) of the bacterial community composition indicated a clear separation between the inoculum and the enriched communities and revealed the existence of two different clusters, separated on the basis of growth on fresh (adaptation phase) versus recalcitrant substrate (stabilization phase) (PERMANOVA, *P* < 0.05, Supplemental Fig. S3; Supplemental Fig. S4).

**Fig. 2:**
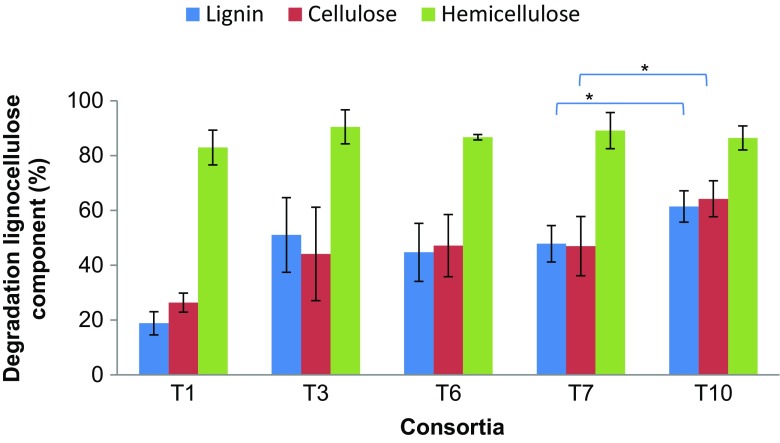
Lignocellulose degradation potential of the communities enriched during the experiment. Percentage reduction of hemicellulose, cellulose, and lignin contents of wheat straw (substrate) comparing with substrate recovered from an not uninoculated control. Explanation: 100% lignin, 100% cellulose, and 100% hemicellulose are equivalent at 18.3% of lignin, 42.5% of cellulose, and 32.5% of hemicellulose in the substrate respectively. Bars refer to standard errors of the mean (*n* = 3)

In contrast, the fungal consortia did not reveal a strong clustering between adaptation and stabilization phases, although they were significantly different from each other (PERMANOVA, *P* < 0.05) (Supplemental Fig. S4). The change in fungal community composition in the stabilization phase was associated with a substantial reduction of the number of bands, confirming the previously described qPCR results, which indicated that, under the applied conditions, fungi are deselected and outcompeted by bacteria.

### Degradation of wheat straw by the microbial consortia

All consortia were found to preferably consume the hemicellulose part of the substrate, which was up to 80% degraded (Fig. 2). None of the selected consortia presented significant differences in hemicellulose degradation (ANOVA, *P* > 0.05). Interestingly, the cellulose part of the wheat straw was degraded to a lower extent, i.e., slightly above 40% (Fig. 2). Comparisons between the consortia across time indicated there was no significant difference in the degradation of hemicellulose, cellulose, and lignin (ANOVA, *P* > 0.05), except at T7 and T10, at which time points significant differences in the degradation of cellulose and lignin were found. The consortia at T10 degraded significantly more cellulose (64.2% ± 6.6) and lignin (61.4% ± 5.7) than those at T7 (cellulose 47% ± 10.8 and lignin 47.8% ± 6.6; ANOVA, *P* < 0.05) (Fig. 2). Comparing the two phases, the consortia from the stabilization phase were able to degrade significantly more lignin than those from the adaptation phase (*T* test, *P* < 0.05).

### Communities structure of the degrading consortia, as determined by 16S rRNA gene-based sequencing

Direct amplicon sequencing performed on a selected number of transfers revealed grossly decreasing bacterial richness values along the transfers. Specifically, for the inocula and the T1, T3, T6, T7, and T10 consortia, the values were 4.84 ± 0.34, 3.49 ± 0.40, 3.40 ± 0.72, 3.14 ± 0.25, 3.41 ± 0.38, and 2.90 ± 0.27, respectively (log OTU number ± standard deviation). Moreover, significant differences in richness were found between the consortia in the adaptation and the stabilization phases, T1, T3 and T6 versus T7 and T10, respectively (ANOVA, *P* < 0.05).

**Fig. 3:**
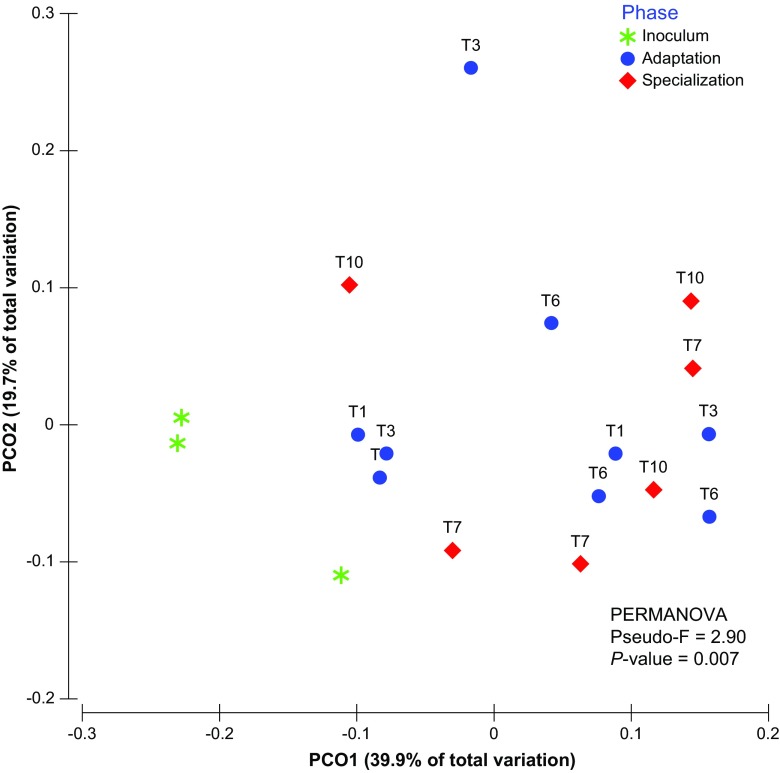
Shifts in bacterial community structure during the adaptation and stabilization phases of the experiment as derived from the 16S rRNA gene sequencing data (V4-V5 region). Principal coordinates analysis (PCoA) of unweighted UniFrac distances for 16S rRNA gene sequencing data of selected enrichment consortia (T1, T3, T6, T7, T10). Fresh substrate (blue circles), used substrate (red diamonds), inoculum (green asterisks). PERMANOVA indicated significant differences between the communities (*P* = 0.007, Pseudo-F = 2.90)

Regarding the bacterial community structures (β-diversities), PCoA of the unweighted UniFrac community distances confirmed the previously described PCR-DGGE results. The data showed that the consortia selected on fresh substrate (adaptation phase, T1, T3, and T6) were markedly different from those selected on recalcitrant substrate (T7 and T10) (Fig. 3). PERMANOVA showed that, indeed, bacterial consortia were significantly different between the adaptation and stabilization phases, as driven by the change in the substrate (*P* < 0.005). This indicated that a clear shift had occurred as a result of the transition from raw to recalcitrant substrate.

**Fig. 4:**
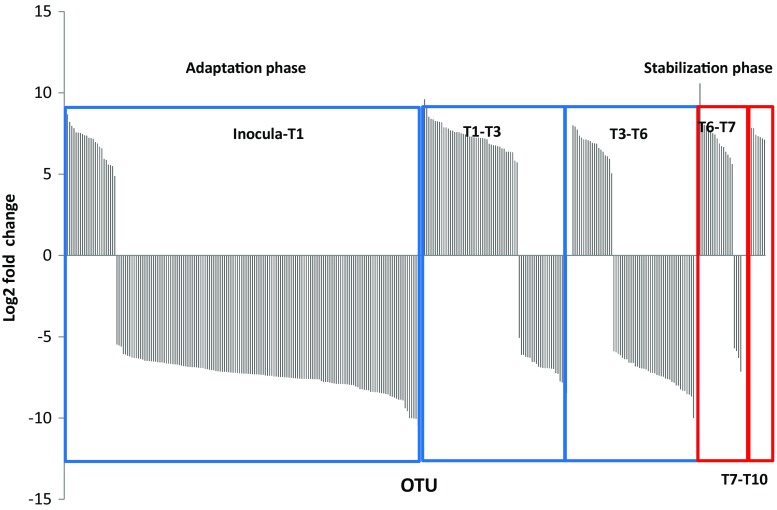
Number of OTUs (log2 fold change) that were positively and negatively influenced in the adaptation and stabilization phases of the experiment. DESeq2 function for *phyloseq* was used to obtain the statistically significant OTUs affected by the enrichment process and the change in substrate composition. Comparisons between selected transfers for adaptation phase included inoculum vs T1, T1 vs T3, and T3 vs T6 (blue squares). In the stabilization phase, the comparison was made between T3 vs T6 and T7 vs T10 (red squares). The adaptation phase shows an important reduction of numbers of OTUs, as indicated by a larger number of bars with negative values especially in the early and late transfers, whereas in the stabilization phase, we observed an increase in the number of OTUs selected—mostly OTUs with positive values were significantly different from one transfer to another

The comparison of the bacterial consortia between the transfers showed that, in the adaptation phase, a large amount of OTUs was significantly affected by the enrichment, leading to a large turnover in community composition and positive selection of OTUs. In contrast, the turnover was lower in the stabilization phase, with relatively few OTUs being negatively affected by the confrontation with the recalcitrant substrate (T7) (Fig. 4). Comparison of the consortia at T7 and T10 (stabilization phase) revealed an increase of abundance of particular OTUs (Fig. 4). Thus, 19 OTUs were differentially selected in the adaptation phase (Table 1) and only five OTUs were positively affected by the change in the substrate during stabilization phase (Table 2). Four OTUs were present in both phases: OTU57506 (affiliated with *Halomonas alkaliphila)*, OTU415 (affiliated with *Algoriphagus winogradskyi* or *ratkowskyi*, OTU358 (*Joostella marina)*, and OTU667 (*Flavobacterium beibuense*).

**Table 1:** Abundant OTUs that were significantly enriched in the adaptation phase (fresh substrate), as determined by 16S rRNA gene sequencing

OTU	Taxonomic affiliation	*Identity (%)	Accession number reference*
OTU57506	*Halomonas meridiana*	99	DQ768627.1
OTU358	*Joostella marina*	99	KP706828.1
OTU667	*Flavobacterium beibuense*	99	KY819115.1
OTU496	*Flavobacterium suzhouense*	98	KM089833.1
OTU665	*Pseudomonas putida*	99	KM091714.1
OTU421	*Stenotrophomonas rhizophila*	99	MF381036.1
OTU176	*Paracoccus seriniphilus*	99	KX453219.1
OTU806	*Nitrosotalea* sp.	99	KJ540205.1
OTU659	*Altererythrobacter* sp.	99	KT325206.1
OTU850	*Halomonas alkaliphila*	99	MF928383.1
OTU49	*Proteinimicrobium ihbtica*	90	AM746627.1
OTU859	*Photobacterium halotolerans*	99	KT354559.1
OTU114263	*Devosia ginsengisoli*	99	KF013197.1
OTU93687	*Bacillus flexus*	99	MF319797.1
OTU253	*Halomonas taeanensis*	95	FJ444986.1
OTU71211	*Rhizomicrobium palustre*	97	NR_112186.1
OTU77552	*Halomonas variabilis*	99	KX351792.1
OTU158296	*Algoriphagus locisalis*	99	NR_115326.1
OTU66912	*Halomonas meridiana*	94	DQ768627.1

*Similarity between the OTU sequence and that of the NCBI entry

**Table 2:** Abundant OTUs that were significantly enriched in the stabilization phase (pre-digested substrate), as determined by 16S rRNA gene sequencing

OTU	Taxonomic affiliation	*Identity (%)	Accession number reference*
OTU358	*Joostella marina*	99	KP706828.1
OTU667	*Flavobacterium beibuense*	99	KY819115.1
OTU415	*Algoriphagus ratkowskyi*	98	KM091714.1
OTU665	*Pseudomonas putida*	99	KM091714.1
OTU57506	*Halomonas meridiana*	99	DQ768627.1

*Similarity between the OTU sequence and that of the NCBI entry

### Degradation of wheat straw by selected strains

In total, 47 bacterial strains were recovered from the consortia at T6 and T10. Most of the strains were isolated from both transfers, except for *Photobacterium halotolerans* A34, *Albirhodobacter marinus* C13, and *Paracoccus seriniphilus* C14, which were recovered only from the adaptation phase (T6) (Table 3). All were identified on the basis of 16S rRNA gene sequencing (Tables 3 and 4). Subsequently, bacterial strains were screened for the production of enzymes able to degrade X-glu, X-cell, X-gal, X-xyl, X-man and X-fuc  (Tables 3 and 4). The data showed that such degradation potential was widespread across the strains. Of the 47 strains tested, only three did not show any enzymatic activity against the selected substrates. These were *Staphylococcus capitis* P1, *Bacillus oleronius* G13, and *Erythrobacter gaetbuli* G57.

**Table 3:** Enzymatic activity of the halotolerant lignocellulose degrading strains recovered from the adaptation phase and their associated OTUs, determined by comparing the complete 16S rRNA gene sequences from the strains with the partial sequences of the same gene obtained by amplicon sequencing. Enzymatic activities were determined by choromogenic essays using the substrates 5-bromo-4-chloro-3-indolyl α-D-glucopyranoside (X-glu), 5-bromo-4-chloro-3-indolyl β-D-cellobioside (X-cell), 5-bromo-4-chloro-3-indolyl α-D-mannopyranoside (X-man), 5-bromo-4-chloro-3-indolyl β-D-galactopyranoside (X-gal), 5-bromo-4-chloro-3-indolyl β-D-xylopyranoside (X-xyl), and 5-bromo-4-chloro-3-indolyl β- fucopyranoside (X-fuc)

Taxonomic affiliation	Enzymatic activity										
*Closest relative	Code	Cover (%)	Identity (%)	X-glu	X-cell	X-gal	X-xyl	X-man	X-fuc	Associated OTU	Accession number
*Albirhodobacter marinus*	C13	100	99	+++	−	−	++	++	−	−	MF619963
*Altererythrobacter indicus*	P4	100	99	+	−	+	−	−	−	OTU659	MF619965
*Altererythrobacter indicus*	G10	100	99	−	−	+++	+	−	−	OTU659	MF619966
*Altererythrobacter indicus*	G19	100	99	−	+++	+++	+++	−	−	OTU659	MF619967
*Arthrobacter nicotianae*	C6	100	99	+++	−	−	+++	+++	−	−	MF619969
*Arthrobacter nicotianae*	M16	100	99	+++	−	−	+++	+++	−	−	MF619968
*Bacillus oleronius*	G13	100	99	−	−	−	−	−	−	−	MF619970
*Demequina aestuarii*	G48	100	99	+++	+	−	−	−	−	−	MF619977
*Demequina aestuarii*	G52	99	99	+++	+	−	+++	−	−	−	MF619978
*Demequina psychrophila*	G33	100	98	+	+++	+++	+++	+	−	−	MF619971
*Demequina psychrophila*	G34	100	98	+++	+++	+++	+++	−	+++	−	MF619972
*Demequina psychrophila*	G35	100	98	+++	+++	+++	+++	−	+++	−	MF619973
*Demequina psychrophila*	G58	100	98	+++	+++	−	+++	−	+++	−	MF619975
*Demequina psychrophila*	G55	100	98	+++	+++	−	+	−	+	−	MF619974
*Demequina psychrophila*	G59	100	98	+++	+++	−	+	−	+++	−	MF619976
*Erythrobacter gaetbuli*	G57	100	96	−	−	−	−	−	−	−	MF619979
*Halonomas alkaliphila*	M10	100	99	+++	−	−	−	−	−	OTU850	MF619983
*Halomonas alkaliphila*	M11	100	99	+++	−	−	−	−	−	OTU850	MF619984
*Microbacterium oleivorans*	G37	99	99	+	+	+++	+	+++	+++	−	MF619992
*Microbacterium oleivorans*	G56	100	98	+	+	+	+	+	+	−	MF619991
*Microbacterium oleivorans*	G46	100	99	+++	+++	+++	+	+++	+++	−	MF619993
*Micrococcus yunnanensis*	G68	100	99	−	−	+	−	−	+++	−	MF619994
*Oceanicola antarcticus*	M45	100	97	−	+	−	−	−	−	−	MF619995
*Paracoccus seriniphilus*	C14	100	99	++	−	++	++	++	−	OTU176	MF619998
*Paracoccus seriniphilus*	M48	100	100	+++	−	+++	+++	+++	−	OTU176	MF619996
*Paracoccus seriniphilus*	G23	100	99	+++	−	+++	−	+++	−	OTU176	MF619997
*Photobacterium halotolerans*	A34	100	99	−	−	+++	−	−	−	OTU589	MF620002
*Photobacterium halotolerans*	M14	100	99	−	−	+++	−	−	−	OTU859	MF619999
*Photobacterium halotolerans*	M15	100	99	−	−	+++	−	−	−	OTU859	MF620000
*Photobacterium halotolerans*	M20	100	99	−	−	+++	−	−	−	−	MF620001
*Pseudorhodobacter incheonensis*	G11	100	99	+++	−	−	−	−	−	−	MF620006
*Sanguibacter inulinus*	G36	100	99	−	−	−	−	−	−	−	MF620007
*Staphylococcus capitis*	P1	100	99	−	−	−	−	−	−	−	MF620008
*Staphylococcus epidermidis*	EG46	100	99	+++	−	−	−	−	−	−	MF620009
*Sarocladium strictum*	HF1	84	96								MF621035

*Closest related species, according to the 16S ribosomal RNA gene sequence. Enzymatic activity: *X-glu*: glucosidases; *X-cell*: cellobiohydrolases; *X-gal*: galactosidases; *X-xyl*: xylosidases; *X-man*: mannosidases;* X-fuc*: fucosidases. Qualitative enzymatic activity detection: (−) no activity ; (+) low activity-light blue; (++) medium activity-medium blue; (+++) high activity- dark intense blue

**Table 4:** Halotolerant lignocellulose degrader strains recovered from the stabilization phase. Potential degradation and matches to closest 16S rRNA gene sequences and associated OTUs. Enzymatic activities were determined by choromogenic essays using the substrates 5-bromo-4-chloro-3-indoly α-D-glucopyranoside (X-glu), 5-bromo-4-chloro-3-indolyl β-D-cellobioside (X-cell), 5-bromo-4-chloro-3-indolyl α-D-mannopyranoside (X-man), 5-bromo-4-chloro-3-indolyl β-D-galactopyranoside (X-gal), 5-bromo-4-chloro-3-indolyl β-D-xylopyranoside (X-xyl) and 5-bromo-4-chloro-3-indolyl β-fucopyranoside (X-fuc)

Taxonomic affiliation	Enzymatic activity										
*Closest relative	Code	Cover (%)	Identity (%)	X-glu	X-cell	X-gal	X-xyl	X-man	X-fuc	Associated OTU	Accession number
*Algoriphagus winogradskyi / ratkowskyi*	G63	100	99		+++	–	+++	–	–	OTU415	MF619964
*Flavobacterium beibuense*	M35	100	99	+++	–	–	–	–	–	OTU667	MF619980
*Flavobacterium beibuense*	M44	100	98	+++	–	–	–	–	–	OTU667	MF619981
*Halomonas meridiana*	G21	100	99	+++	–	–	–	–	–	OTU57506	MF619985
*Halomonas neptunia*	M8	100	99	+++	–	+++	–	–	–	–	MF619986
*Halomonas venusta*	M9	97	99	+++	+	–	–	–	–	–	MF619987
*Joostella marina*	G54	100	99	+++	–	+++	+	+++	–	OTU358	MF619989
*Joostella marina*	G65	100	99	+++	+++	+++	–	–	+++	OTU358	MF619990
*Joostella marina*	ME32	100	99	+++	–	+++	+++	+++	–	OTU358	MF619988
*Pseudomonas sabulinigri*	G20	100	99	+	–	–	–	–	–	–	MF620005
*Pseudomonas sabulinigri*	M38	100	99	+	–	–	–	–	–	–	MF620004
*Pseudomonas sabulinigri*	M7	100	99	+++	–	–	–	–	–	–	MF620003

*Closest related species, according to 16S ribosomal RNA gene sequence. Enzymatic activity: *X-glu:* glucosidases, *X-cell:* cellobiohydrolases, *X-gal:* galactosidases, *X-xyl:* xylosidases, *X-man:* mannosidases, *X-fuc:* fucosidases. Qualitative enzymatic activity detection: (−) no activity , (+) low activity—light blue, (++) medium activity—medium blue, (+++) high activity—dark intense blue

**Fig. 5:**
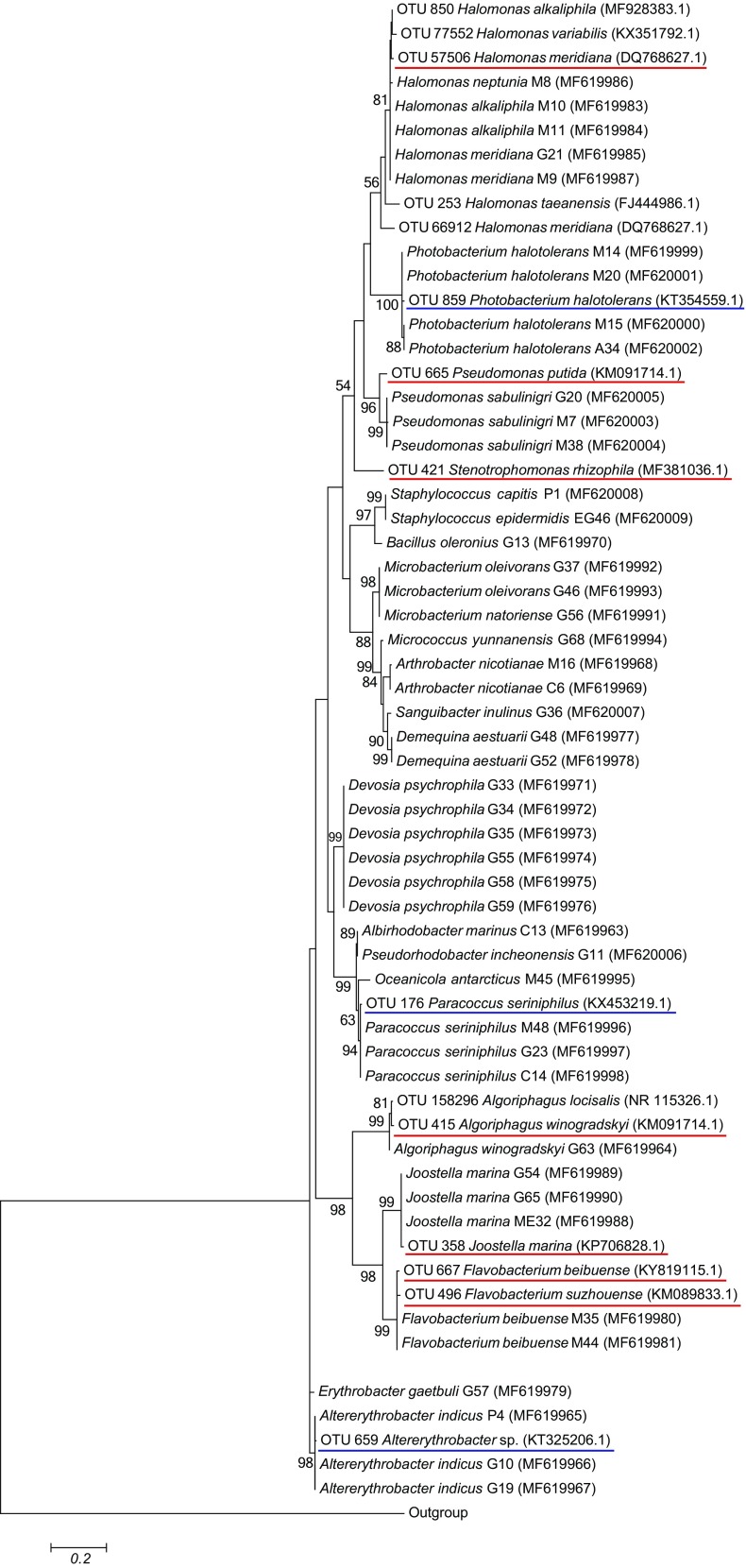
Phylogenetic affiliation of 16S rRNA gene sequences of isolated strains and sequenced OTUs. Neighbor Joining tree based on the 16S rRNA gene sequences (V4-V5 region) from bacterial strains and from the significant abundant OTUs at the end of the adaptation phase (T6) and stabilization phase (T10). For the adaptation phase, underlined in blue are OTU850 *H. alkaliphila* (99%, MF928383.1), OTU859 *P. halotolerans* (99%, KT354559.1), OTU176 *P. seriniphilus* (99%, KX453219.1), and OTU659, *Altererythrobacter* sp. (99%, KT325206.1). For the stabilization phase, underlined in red, OTU57506 *H. meridiana* (99%), OTU665 *P. putida* (99%), OTU415 *A. winogradskyi*/*ratkowskyi* (98%), OTU358 *J. marina* (99%, DQ768627.1), and OTU667 *F. beibuense* (99%, KY819115.1). Between brackets: % of identity, reference accession number. The 16S rRNA gene sequence from *Methanocaldococcus jannaschii* was used as outgroup. Bar indicated divergence scale (0.2 = 20%)

By aligning the 16S rRNA gene sequences recovered from the isolated bacteria with those of the OTUs obtained by direct sequencing (Fig. 5), we were able to pinpoint the strains that were highly abundant in the consortia (Tables 3 and 4). In the adaptation phase, nine strains were closely related to four enriched OTUs (Table 3). Those were affiliated with *Halomonas alkaliphila* (M10 and M11), *Photobacterium halotolerans* (A34, M14, M15, and M20), *Paracoccus seriniphilus* (C14 and M48), and *Altererythrobacter indicus* (P4, G10, and G19). In the stabilization phase, seven strains were closely related to four enriched OTUs (Table 4): *Halomonas meridiana* M11, *Algoriphagus winogradskyi* G63, *Jootella marina* (G54, G65, and ME32), and *Flavobacterium beibuense* (M35 and M44). Finally, we recovered two strains affiliated with *Pseudomonas sabulinigri* G20 and M7; however, these did not match the OTU665 (affiliated with *Pseudomonas putida*) (Fig. 5)*.*

Tables 3 and 4 show details of enzyme production by the strains. On the one hand, strains isolated from the adaptation phase yielded not only most of the tested hydrolytic activities, but also showed the highest activities. Remarkably, the strains affiliated with *Microbacterium oleivorans* (G37, G46) and *Devosia psychrophila* (G33-G35) revealed the production of five or even six hydrolytic enzymes (Table 3). On the other hand, strains isolated from the recalcitrant substrate were less versatile than those isolated from fresh substrate, as evidenced by the lower number of enzymatic activities (three out of six tested). Only the strains affiliated with *J. marina* (ME32, G54, and G65) presented the capacity to produce at least four hydrolytic enzymes with high activity.

### Fungal strains from the stabilization phase

As mentioned before, the change in the substrate had an important effect on the fungal community. Only one fungal strain was obtained from the adaptation phase (Table 3). It was affiliated with *Sarocladium strictum* HF1 and was obtained from all triplicate plates. It was, however, not possible to recover any fungal strain from the stabilization phase. Despite the bands observed in DGGE (based on the ITS1 region) in the stabilization phase, we observed a sharp decline in fungal abundance—as determined by qPCR targeting the same region (Fig. 1b)—at the end of the experiment (T10), which probably hindered isolation.

## Discussion

In this study, we produced and characterized microbial consortia—potential sources of lignocellulose degraders and their enzymes—that were capable of degrading wheat straw under high-salt concentrations, a condition often established by particular lignocellulose pretreatment steps. Thus, our selected halotolerant microbial consortia represent a clear prospect of lignocellulose degradation under saline conditions, as they may either be used to directly unlock lignocellulose biomass or to produce halotolerant lignocellulolytic enzymes. The latter application may eliminate the expensive washing steps, reducing costs.

### Saline conditions favor bacterial over fungal degraders

Changes in wheat straw content can considerably affect the composition of microbial communities growing on it. Here, in particular, fungal densities decreased significantly in the stabilization phase (when recalcitrant substrate was used), hindering our ability to isolate fungal strains. It is generally believed that fungi are ubiquitous and capable of occupying virtually every ecological niche as a result of their ability to degrade a suite of organic compounds such as complex biological polymers. They may also play roles in degrading lignocellulose in marine environments (Richards et al. 2012), where the major factors affecting their diversities are salt concentration and temperature (Fuentes et al. 2015). For instance, it has been shown that several fungal strains recovered from mangrove systems are capable of growing on wood under high-salt conditions (Arfi et al. 2013). In our study, the only isolated fungal strain—*Sarocladium strictum*, previously known as *Acremonium strictum* (Summerbell et al. 2011)—is likely well adapted to saline environments, as it was previously isolated from a marine ecosystem (Fuentes et al. 2015). Here it originated from a salt-marsh soil inoculum. It was, however, only recovered in the adaptation phase, declining in density (to below the detection limit) in the stabilization part of the experiment. Although we cannot pinpoint the exact reason for the observed decline in fungal density (considering that both temperature and salt concentration remained constant in our experiment), we argue that this reduction could be explained by nitrogen depletion in the recalcitrant substrate, consistent with the findings by Meidute et al. (2008). Thus, the impossibility to isolate fungal strains from the specialized consortia could be related to a very strong nutritional demand under the prevailing conditions, leading to a decline in density that hindered isolation. Additionally, pH could be an important factor affecting the viability of the fungi in our system. During the cultivation, the pH decreased slightly from 7.2 to 6.8, which is higher than the optimal pH for fungal growth (between pH 2.2 and 6.5; Matthies et al. 1997). Moreover, the maintenance of the almost neutral pH along the incubation suggested a low production of organic acids (which indicates that massive fermentation did not occur). The maintenance of prevailing aerobic conditions in the culture probably incited mostly oxidative phosphorylation processes. Thus, in the system (an agitated saline environment with a recalcitrant source of carbon and energy), bacteria probably had a main role in the degradation process.

The dominance of bacteria over fungi in our halotolerant lignocellulose grown consortia is interesting, as previous studies, performed under non-saline conditions, suggested that fungal communities have a relevant participation in lignocellulose degradation, even working in liquid and agitated systems. For example, Brossi et al. (2015) found that *C. ligniaria* (strains WS1, WS2, SG8) had a significant role in the degradation of diverse lignocellulose feedstocks, while Jiménez et al. (2013) found the same organism (strain 2w1F) played a crucial role in the decomposition of wheat straw in presence of 5-hydroxymethylfurfural. In both cases, the dilution-to-stimulation approach was used for the selection of the degrader communities.

### Substrate quality greatly impacts community composition

The findings in this study clearly indicate that substrate quality and composition direct the structure of microbial consortia (Simmons et al. 2014; Brossi et al. 2015), which developed to degrade  either fresh (adaptation phase) or previously- degraded (recalcitrant) lignocellulose substrate (stabilization phase). Whereas the fresh substrate allowed the selection of a more generalist degrading community, composed of very specific bacterial and fungal strains, the recalcitrant substrate  selected for more specialized, mostly bacterial, species. Interestingly, all replicates of the enrichment process gave fairly similar patterns, in terms of consortium development, both quantitatively (viz the bacterial and fungal abundance values) and with respect to the bacterial community structures, demonstrating the robustness of our findings. We thus posit here that a consistent selection of microorganisms with progressively higher abilities to grow (jointly) on the substrate had taken place. In the consortia, bacteria were quantitatively by far more important than fungi, and so we placed a greater focus on the bacterial part of the resulting consortia. This bacterial dominance was even exacerbated by the shift to a more recalcitrant substrate after T6.

### Wheat straw degradation and potential involvement of identified strains

On the basis of all our data, we depict the degradation of wheat straw under saline conditions to proceed in a sequential manner, with different microbes being dominant in a spatiotemporally explicit form. The wheat straw, being recalcitrant, poses clear obstacles to degradation. The main hurdles are the presence of crystalline cellulose and the bonding between lignin and hemicellulose (shielding the latter component from access by key enzymes). We briefly discuss these issues in the paragraphs below.

Crystalline cellulose is highly recalcitrant to chemical and biological hydrolysis due to the strongly linked chains of cellodextrins. The decomposition of crystalline cellulose, for example filter paper, requires the production of specific cellulases. In our consortia, *Joostella marina* (OTU358) and *Flavobacterium beibuense*(OTU667) may have had a main role in cellulose degradation, as we observed increases in their abundances in the stabilization phase. Also, the consortia from this phase displayed higher cellulose degradation capacities than consortia from the adaptation phase. Both *J. marina* (OTU358) and *F. beibuense *(OTU667) belong to the *Flavobacteriaceae* (Bernardet et al. 2002)*.* Members of this family have been isolated from soil, sediment and marine/saline environments, and they have been typically associated with decomposition of complex polysaccharides (Lambiase 2014). Some species in the family degrade soluble cellulose derivatives such as carboxymethyl-cellulose. However, since enzymes other than cellulases can degrade this compound, this does not demonstrate that these species are cellulolytic. *J. marina* probably has an important role in the degradation of recalcitrant regions of lignocellulose substrate, as it is capable to grow on complex hydrocarburic substrate (Rizzo et al. 2015). The organism is strictly aerobic and can grow in up to 15% NaCl, with glucose, arabinose, mannose, and cellobiose as single carbon and energy sources. Additionally, it has been reported to be positive for α-glucosidase, β-glucosidase, β-galactosidase, and α-mannosidase production (Stackebrandt et al. 2013). In our final consortia, *J. marina* could be associated with the degradation of the crystalline cellulose in the wheat straw. However, more studies are needed to demonstrate such cellulolytic capability. Currently, this characteristic is restricted to members of the *Cytophagaceae* family (Bernardet et al. 2002). Additionally, the *Flavobacterium* species found in this study (*F. beibuense* OTU667 and *Flavobacterium suzhouense* OTU496) may be only associated with the degradation of amorphous cellulose, which is readily digestible. These organisms can degrade soluble cellulose such as hydroxymethylcellulose and cellodextrine (Lambiase 2014).

Regarding lignin degradation or bond hydrolysis, the increasing abundance of *Pseudomonas* species (*P. putida* OTU665 and *P. sabulinigri* G20, M38, and M7*)* in the stabilization phase suggest a role for these organisms in the relevant transformation steps, such as the degradation of recalcitrant regions of the substrate like residual hemicellulose linked to lignin structures. *Pseudomonas* species stand out as having a great potential capacity for lignin degradation (Beckham et al. 2016). For instance, in a recent study, *P. monteilli* and *P. plecoglossicida* were enriched from mature vegetal compost. These organisms were found to degrade a large amount of lignin-related compounds (Ravi et al. 2017). In another study, Salvachúa et al. (2015) isolated *P. putida*, *Rhodococcus jostii*, and *Acinetobacter* sp. ADP1, all of which were able to depolymerize and catabolize high-molecular-weight lignin (Salvachúa et al. 2015).

The most labile part of the substrate, hemicellulose, was probably mainly attacked by *H. meridiana* (OTU 57506) and related species*.* Their decreased abundance in the stabilization phase could indicate that the hemicellulose part of the substrate was largely depleted. *H. meridiana* belongs to the class *Gammaproteobacteria*. It is a facultatively halotolerant organism capable of growth in NaCl concentrations between 0.1 and 32.5% (*w*/*v*). It is mostly found in marine environments (Octavia and Lan 2014). A recent study suggested that *H. meridiana* has great potential for biotechnology applications, as a producer of extracellular enzymes adapted to salinity (Yin et al. 2015).

Finally, *Algoriphagus winogradskyi/ratkowskyi* G63, belonging to the *Cytophagaceae,* could be involved in the degradation of both the hemicellulose and cellulose regions of the substrate. A genetic analysis of *Algoriphagus* sp. PR1 demonstrated its high capacity of polysaccharide degradation, as large numbers of genes encoding glycoside hydrolases, polysaccharide lyases, carbohydrate esterases, and glycosyltransferases were found (Alegado et al. 2011). Previous reports indicated that related strains cannot degrade filter paper (Lambiase 2014), however our strains were not yet tested for such activity.

Although the contribution of fungi to the degradation process seems to be restricted to the adaptation phase, previous reports have demonstrated the biotechnological application of *Sarocladium strictum*. Interestingly, this was our only isolated fungal strain, and one may envision a role for it in the production of cellulases direct from infested lignocellulose feedstock (Goldbeck et al. 2013). Also, a gene for gluco-oligosaccharide oxidase from this species has been engineered (high catalytic activity and lowsubstrate inhibition) for application in industrial plant polysaccharide degradation (Domon et al. 2013). Definitely, more studies are necessary on *S. strictum* to examine all its degradation capacities, although it might be restricted to conditions with high nutrient supply.

In conclusion, the construction of microbial consortia able to grow on wheat straw as a carbon and energy source under saline conditions offers access to salt-adapted or salt-tolerant enzymes (haloenzymes) that enable the development of processes under saline conditions. It is assumed that the selected organisms harbor the potential to naturally produce such salt-adapted enzymes, which are applicable in a bioprocess with raised NaCl levels. We propose that the key members of our consortia yield very interesting salt-tolerant enzymes for bioengineering, as follows: (1) *J. marina* (G54, G65, ME32): production of carbohydrate esterases, (2) *F. beibuense* (M35, M44): production of cellulases, (3) *P. sabulinigri* (G20, M38, M7): production of ligninases, and (4) *H. meridiana* (G21): production of hemicellulases. A key issue here is the precise combination of enzymes that is required to establish an efficient “saline bioprocess”. Potentially, such an enzyme mixture is made on the basis of the organisms as described here.

## Electronic supplementary material

ESM 1 (PDF 587 kb)
