# Supplementary material for: Halotolerant microbial consortia able to degrade highly recalcitrant plant biomass substrate
Source: Appl Microbiol Biotechnol. 2018 Feb 3;102(6):2913–27. doi: 10.1007/s00253-017-8714-6 (PMC5847192; doi:10.1007/s00253-017-8714-6)
Supplement: Supplementary file 1 — (PDF 587 kb) [file 253_2017_8714_MOESM1_ESM.pdf]

Applied Microbiology and Biotechnology

"Halotolerant microbial consortia able to degrade highly recalcitrant plant biomass substrate"

Larisa Cortes-Tolalpa*, Justin Norder , Jan Dirk van Elsas Joana Falcao Salles.

Genomics Research in Ecology and Evolution in Nature, Groningen Institute for Evolutionary Life Sciences, University of Groningen, Groningen, The Netherlands[a]

*Corresponding author: Larisa Cortes-Tolalpa, l.cortes.tolalpa@gmail.com, Nijenborgh 7, 9747 AG, Groningen, The Netherlands, +31 50 363 2236.

Supplemental Table S1. Primers used for paired-end 16S rRNA gene sequencing on the Illumina MiSeq platform (amplicon sequencing).

SampleID	Reverse primer	Forward primer	Source	Description
Inoculum_1	AGTACGCTATTGYCAGCMGCCGCGGTA	ACGCTCGACACCGYCAATTYMTTTRAGTTT	soil	Soil inoculum
Inoculum_2	ATAGAGTACTTGYCAGCMGCCGCGGTA	AGACGCACTCCCGYCAATTYMTTTRAGTTT	soil	Soil inoculum
Inoculum_3	TACAGATCGTTGYCAGCMGCCGCGGTA	TAGTATCAGCCCGYCAATTYMTTTRAGTTT	soil	Soil inoculum
T1_1	TAGTGTAGATTGYCAGCMGCCGCGGTA	TGATACGTCTCCGYCAATTYMTTTRAGTTT	soil	Transfer 1, flask 1
T1_2	TCTATACTATTGYCAGCMGCCGCGGTA	ATACGACGTACCGYCAATTYMTTTRAGTTT	soil	Transfer 1, flask 2
T1_3	CGCAGTACGATGYCAGCMGCCGCGGTA	ACATACGCGTCCGYCAATTYMTTTRAGTTT	soil	Transfer 1, flask 3
T3_1	CGTACAGTCATGYCAGCMGCCGCGGTA	ACTACTATGTCCGYCAATTYMTTTRAGTTT	soil	Transfer 3, flask 1
T3_2	CGTACTCAGATGYCAGCMGCCGCGGTA	ACTGTACAGTCCGYCAATTYMTTTRAGTTT	soil	Transfer 3, flask 2
T3_3	CTACGCTCTATGYCAGCMGCCGCGGTA	AGACTATACTCCGYCAATTYMTTTRAGTTT	soil	Transfer 3, flask 3
T6_1	TAGTCGCATATGYCAGCMGCCGCGGTA	ATAGAGTACTCCGYCAATTYMTTTRAGTTT	soil	Transfer 6, flask 1
T6_2	TGTCACACGATGYCAGCMGCCGCGGTA	TCGATCACGTCCGYCAATTYMTTTRAGTTT	soil	Transfer 6, flask 2
T6_3	TGTCGTCGCATGYCAGCMGCCGCGGTA	TCGCACTAGTCCGYCAATTYMTTTRAGTTT	soil	Transfer 6, flask 3
T7_1	ACACATACGCTGYCAGCMGCCGCGGTA	TCTAGCGACTCCGYCAATTYMTTTRAGTTT	soil	Transfer 7, flask 1
T7_2	ACATGACGACTGYCAGCMGCCGCGGTA	TGACGTATGTCCGYCAATTYMTTTRAGTTT	soil	Transfer 7, flask 2
T7_3	ACGTCTCATCTGYCAGCMGCCGCGGTA	ACAGTATATACCGYCAATTYMTTTRAGTTT	soil	Transfer 7, flask 3
T10_1	ACTCGCGCACTGYCAGCMGCCGCGGTA	ACTAGCAGTACCGYCAATTYMTTTRAGTTT	soil	Transfer 10, flask 1
T10_2	ATATAGTCGCTGYCAGCMGCCGCGGTA	CGCGTATACACCGYCAATTYMTTTRAGTTT	soil	Transfer 10, flask 2
T10_3	CACGTAGATCTGYCAGCMGCCGCGGTA	CGTACTCAGACCGYCAATTYMTTTRAGTTT	soil	Transfer 10, flask 3

Reference:

Caporaso JG, Lauber CL, Walters WA, Berg-Lyons D, Huntley J, Fierer N, Owens SM, Betley J, Fraser L, Bauer M, Gormley N, Gilbert JA, Smith G, Knight R (2012) Ultra-high-throughput microbial community analysis on the Illumina HiSeq and MiSeq platforms. ISME J 6:1621–1624. doi: 10.1038/ismej.2012.8

Supplemental Table S2. Cellulose, hemicellulose (xylan) and lignin mixtures used to obtain the prediction model.

Ternary mixtures	Lignin (%)	Cellulose (%)	Hemicellulose (%)
A	100	0	0
B	0	100	0
C	0	0	100
D	50	25	25
E	25	50	25
F	25	25	50
G	75	25	0
H	25	75	0
I	25	0	75
J	0	25	75
K	33	33	33
L	72	0	25
M	0	75	25

ESM 1. Isolation procedure of bacterial and fungal strains.

Isolation of bacterial and fungal strains. Serial dilutions were done in saline MSM (25g/L NaCl) and 100 µL aliquots of the 10^{-1}- to 10^{-3} and 10^{-7} to 10^{-9} dilutions, for fungi and bacteria, respectively, were spread on the surface of each of the media. Morphological differences of the colonies were used to select the isolates, which were streaked to purity and then preserved at -80°C (in LB broth with 20% glycerol). To obtain a presumptive identification, genomic DNA was produced by using the UltraClean® Microbial DNA isolation kit (MoBio®). For bacteria, we first de-replicated the isolates based on an ERIC-PCR using primers ERIC1R and ERIC2 (Versalovic et al. 1994; Puentes-Téllez and Elsas 2014). The ERIC-PCR cluster analyses were performed using GelCompar software. Bacterial 16S rRNA genes of representative strains for each ERIC group were amplified using 10 ng of DNA and primers B8F and U1406R (Taketani et al. 2010). For fungal strains (pretreated with liquid nitrogen), genomic DNA was obtained using UltraClean® Microbial DNA isolation kit (MoBio®).

References

Puentes-Téllez PE, Elsas JD van (2014) Sympatric metabolic diversification of experimentally evolved *Escherichia coli* in a complex environment. Antonie Van Leeuwenhoek 106:565–576. doi: 10.1007/s10482-014-0228-y

Taketani RG, Franco NO, Rosado AS, van Elsas JD (2010) Microbial community response to a simulated hydrocarbon spill in mangrove sediments. J Microbiol Seoul Korea 48:7–15. doi: 10.1007/s12275-009-0147-1

Versalovic J, Schneider M, De Bruijn FJ, Lupski JR (1994) Genomic fingerprinting of bacteria using repetitive sequence-based polymerase chain reaction. Methods Mol Cell Biol 5:25–40.

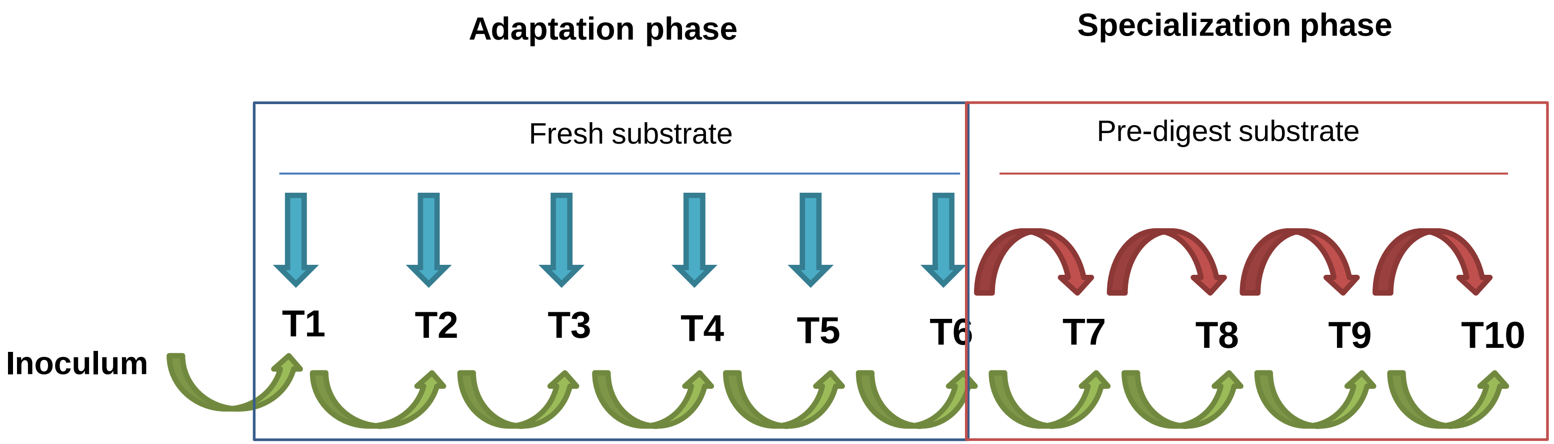

Supplemental Fig. S1 Schematic representation of the experimental approach, conducted in 2 phases, in which microbial communities from soils with high salt concentration were used as inoculum to select for halotolerant lignin- degrading microbial consortia. Adaptation phase: during the first 6 transfers was used fresh lignocellulose substrate (blue arrow). Stabilization phase: from transfers 6 the substrate was recover and used the sub-sequential transfer until T10 (red arrow).

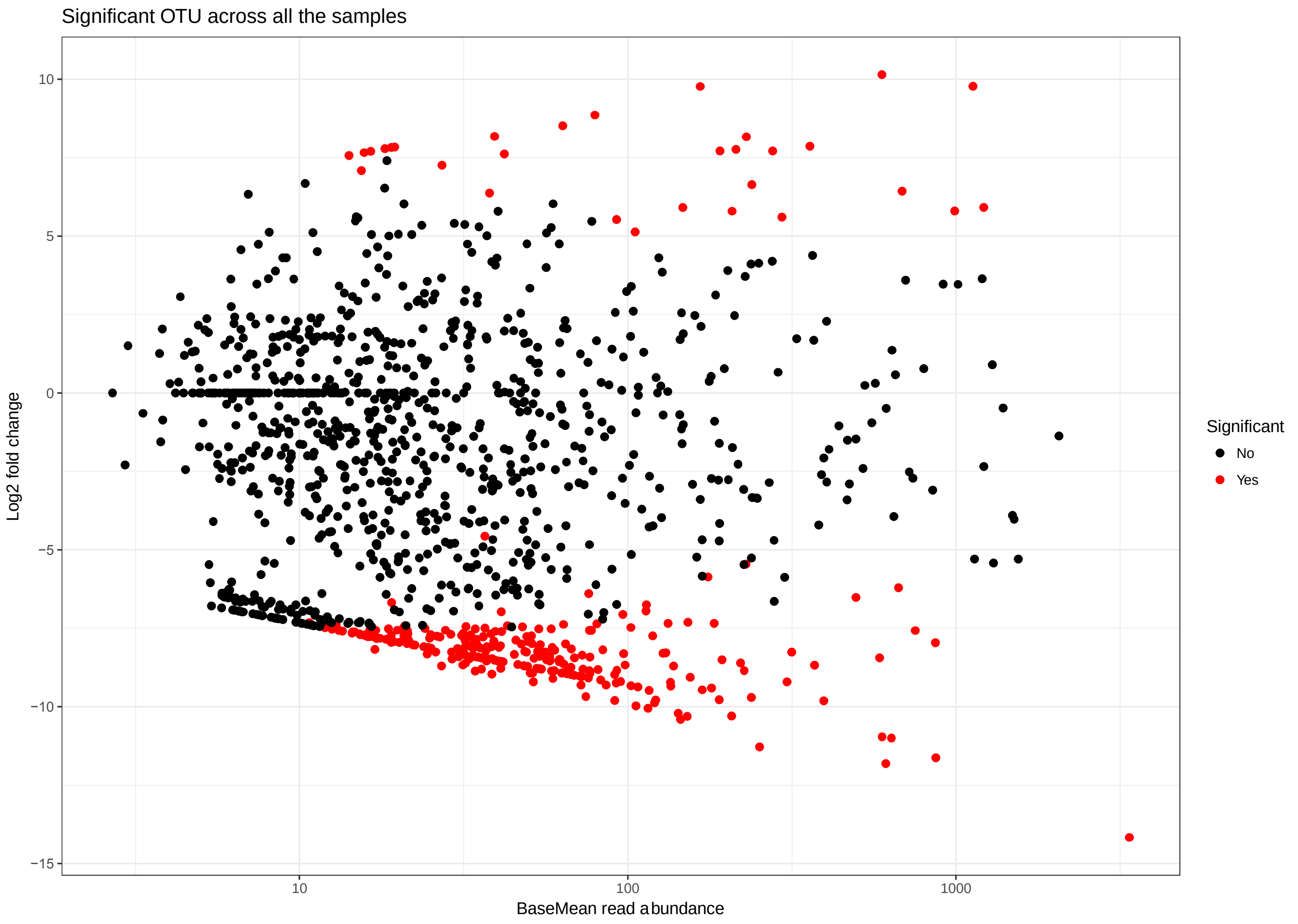

Supplemental Fig. S2. Identification of OTUs that changed significant in abundance (Y-axis: log2 fold change). DESeq2 function for *phyloseq* was used to obtain the statistically significant OTU affected by the enrichment process. OTUs with significant increase in red dots, OTU without significant increase in black dots.

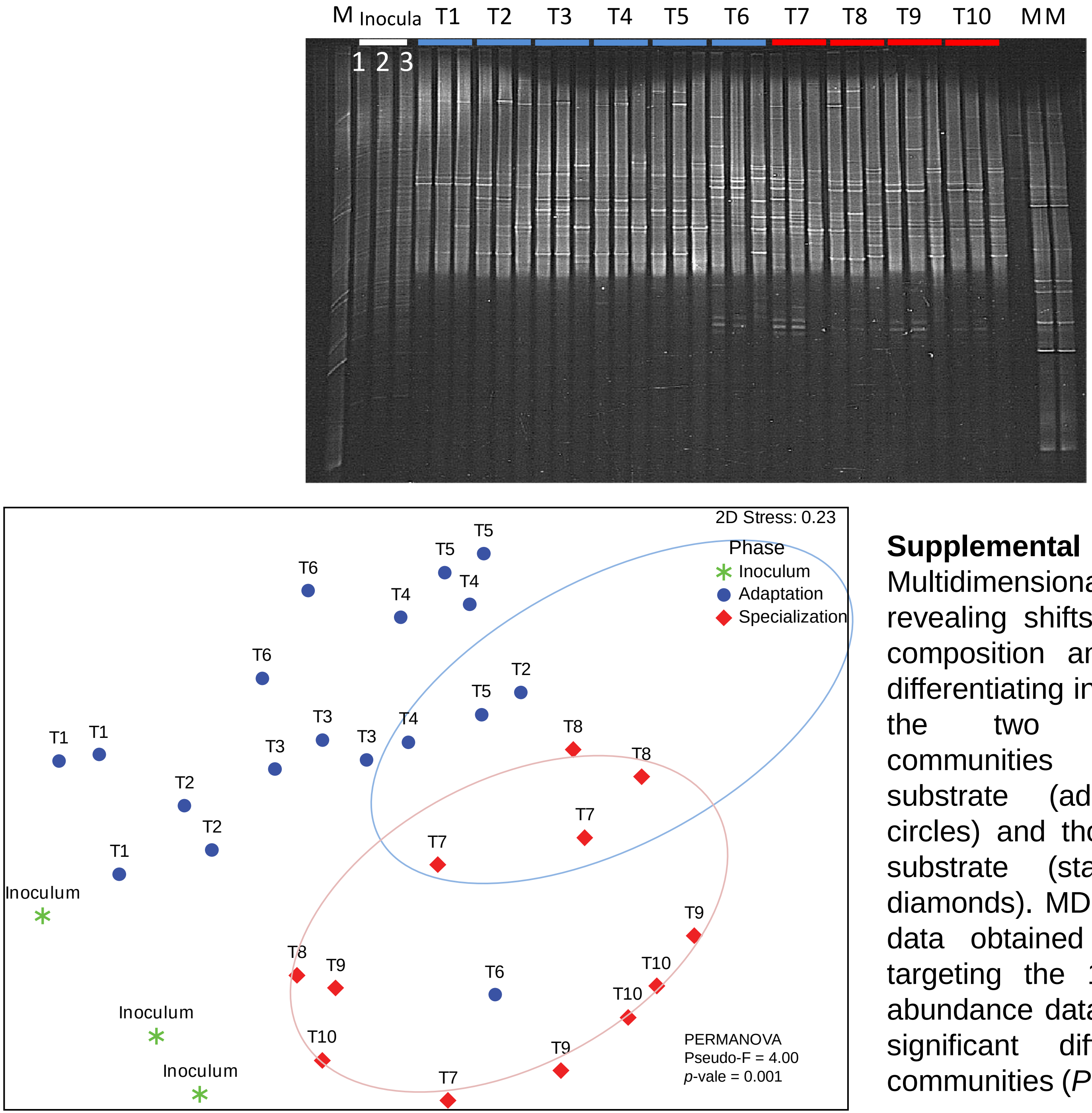

Supplemental Fig. S3. Multidimensional scaling (MDS) revealing shifts in bacterial community composition and well-defined clusters differentiating inoculum (green star), and the two enrichment phases: communities selected with fresh substrate (adaptation phase, blue circles) and those selected with waste substrate (stabilization phase, red diamonds). MDS was constructed using data obtained from the PCR-DGGE targeting the 16S rRNA gene, using abundance data. PERMANOVA indicate significant difference between the communities (*P*= 0.001, Pseudo-F= 4).

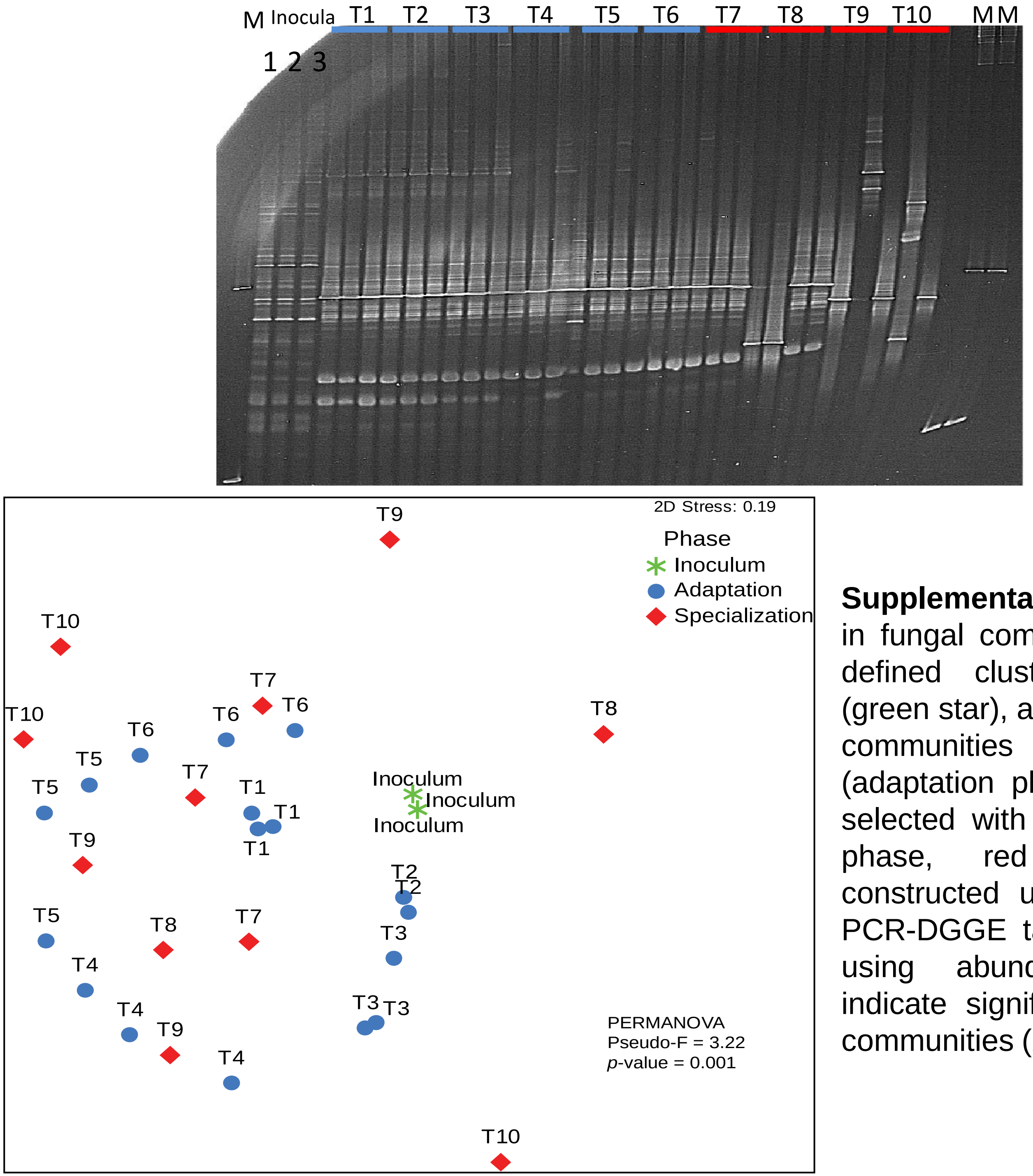

Supplemental Fig. S4. MDS revealing shifts in fungal community composition and well-defined clusters differentiating inoculum (green star), and the two enrichment phases: communities selected with fresh substrate (adaptation phase, blue circles) and those selected with waste substrate (stabilization phase, red diamonds). MDS was constructed using data obtained from the PCR-DGGE targeting the 16S rRNA gene, using abundance data. PERMANOVA indicate significant difference between the communities (*P*= 0.001, Pseudo-F= 4).
